# Anti-Inflammatory and Tissue Regenerative Effects of Topical Treatment with Ozonated Olive Oil/Vitamin E Acetate in Balanitis Xerotica Obliterans

**DOI:** 10.3390/molecules23030645

**Published:** 2018-03-13

**Authors:** Monica Currò, Tiziana Russo, Nadia Ferlazzo, Daniela Caccamo, Pietro Antonuccio, Salvatore Arena, Saveria Parisi, Patrizia Perrone, Riccardo Ientile, Carmelo Romeo, Pietro Impellizzeri

**Affiliations:** 1 Department of Biomedical and Dental Sciences and Morphofunctional Imaging, University of Messina, Via Consolare Valeria, 98123 Messina, Italy; moncurro@unime.it (M.C.); nadiaferlazzo@email.it (N.F.); dcaccamo@unime.it (D.C.); 2 Department of Human Pathology of Adult and Childhood “Gaetano Barresi”, University of Messina, Via Consolare Valeria, 98123 Messina, Italy; russotiziana82@gmail.com (T.R.); pantonuccio@unime.it (P.A.); salvatore.arena@gmail.com (S.A.); parisi.saveria@libero.it (S.P.); perronepatriziaa@gmail.com (P.P.); romeoc@unime.it (C.R.); impellizzerip@unime.it (P.I.)

**Keywords:** balanitis xerotica obliterans, antioxidant therapy, ozonated olive oil, vitamin E acetate, inflammation, tissue regeneration, HIF-1 alpha

## Abstract

Balanitis xerotica obliterans (BXO) is a chronic inflammatory skin disorder, considered the male genital variant of lichen sclerosus. Anti-inflammatory drugs are commonly used in BXO. We evaluated the effects of an innovative formulation of ozonated olive oil with vitamin E acetate (OZOILE^®^) on the inflammatory status and tissue remodeling in male children with BXO. The mRNA transcripts of proteins involved either in inflammation or in dynamics of tissue regeneration were analyzed by quantitative real-time PCR, in foreskins affected by BXO removed from patients untreated or treated with OZOILE^®^ cream for 7 days before circumcision. We found a significant reduction in mRNA levels of IL-1β, TNF-α, INF–γ, transglutaminase 2 and NOS2 in foreskins treated with OZOILE^®^ in comparison to untreated ones (*p* < 0.001). No significant differences were observed in NF-κB activation in the specimens obtained from treated and untreated patients. Hence, OZOILE^®^ treatment up-regulated hypoxia-inducible factor (HIF)-1alpha, vascular endothelial growth factor (VEGF) and E-cadherin gene expression (*p* < 0.001). The treatment with OZOILE^®^ showed effective results in children affected by BXO by reducing the inflammatory process and stimulating mechanisms for tissue regeneration of the foreskin. A randomized clinical trial on a large number of children affected by BXO might be useful to verify the efficacy of topical treatment with OZOILE^®^.

## 1. Introduction

Balanitis xerotica obliterans (BXO), a genital form of lichen sclerosus et atrophicus (LSA) in males, is a chronic inflammatory disease of unknown aetiology that may affect the foreskin, glans, frenulum, and meatus or urethra. The term describes the anatomic location, clinical appearance and histological spectrum associated with the diseases [1,2].

First described by Sthumer [3] as a post circumcision phenomenon, BXO is generally considered responsible for most cases (80 to 90%) of acquired phimosis [4]. Circumcision can be curative for disease confined to the foreskins or glans. Complications, from 2 to 40%, include fusion of the prepuce to the glans, meatal stenosis and urethral strictures. The disease can be associated with a risk of malignant transformation [5].

BXO may be diagnosed clinically with whitish, porcelain-like sclerotic scarring of the distal portion of the foreskin causing progressive phimosis and typically appearing as a whitish ring [4]. Although BXO can be detected clinically, the diagnosis is based on histopathological examination. The aetiology of this disease is not clear: it is believed to be caused through both humoral and cell mediated autoimmunity and has been associated with atopy and other autoimmune conditions [1,4].

Circumcision is the treatment of choice, but topical use of several drugs has been used before or after surgical treatment [6]. The British Association of Dermatologists guideline recommends the treatment of patients affected by BXO with a highly potent corticosteroid [7]. A meta-analysis of randomized trials carried out in adults and children on topical treatment showed that clobetasol proprionate represents the most effective steroid in the treatment of BXO in terms of reduction of symptoms and inflammatory status, and clinical remission [8]. A Cochrane review described the use of clobetasol propionate as the most common and efficient treatment in adults, but not enough data were available to make specific recommendation for male children [9]. In addition, clobetasol proprionate can have several side effects, such as skin atrophy, hypopigmentation, contact lesions, and local fungine infections. Therefore, in some studies, the effectiveness of other drugs has been compared with clobetasol propionate. A cream with testosterone and hydrotestosterone showed a lower efficacy than clobetasol propionate [8]. Goldstein et al. [10] evaluated the response to pimecrolimus cream, a calcineurin inhibitor that inhibits the proliferation of T cells after stimulation of both specific and non-specific antigens, showing less efficacy than clobetasol propionate.

For almost 40 years, the ozone, a highly unstable gas with strong oxidizing capacity, has been used for medical treatment. Ozone effects have been widely studied; in particular, its strong oxidizing capacity has been tested in order to verify its disinfectant and sanitizing properties [11,12,13]. Ozonides are a class of chemical compounds in which ozone is stabilized by the reaction with unsaturated fatty acids of oils. Ozonides represent an alternative to pharmacological therapy [14] and they have been used as topical formulation with germicidal properties [15,16]. Ozonated olive oil has been reported to be non-toxic, accelerate wound healing and exert anti-inflammatory effects [14]. In particular, in contact with skin and mucosae, ozonated olive oil, in environments characterized by a protonic increase, such as ischemic, hypoxic, or damaged tissues, releases molecular oxygen driving the production of radical species with generation of moderate oxidative stress. These effects promote the liberation of growth factors, activation of local antioxidant mechanisms, and tissue repair [14,17].

To date, some studies have shown the therapeutic use of ozonated oil on skin disease [18]. Recently, a new cream composed of ozonated olive oil with vitamin E acetate (OZOILE^®^), obtained by the gasification of biological olive oil with ozone, has been proposed for treatment of mucosal diseases and healing process of the skin. 

In this observational study carried out in male children with BXO, for the first time, we evaluated the beneficial effects of topical treatment with OZOILE^®^ cream, in term of anti-inflammatory action and tissue regeneration.

## 2. Results

Foreskin samples obtained from BXO patients untreated or treated with OZOILE^®^ were examined for the mRNA levels of several markers of inflammation.

**Figure 1:**
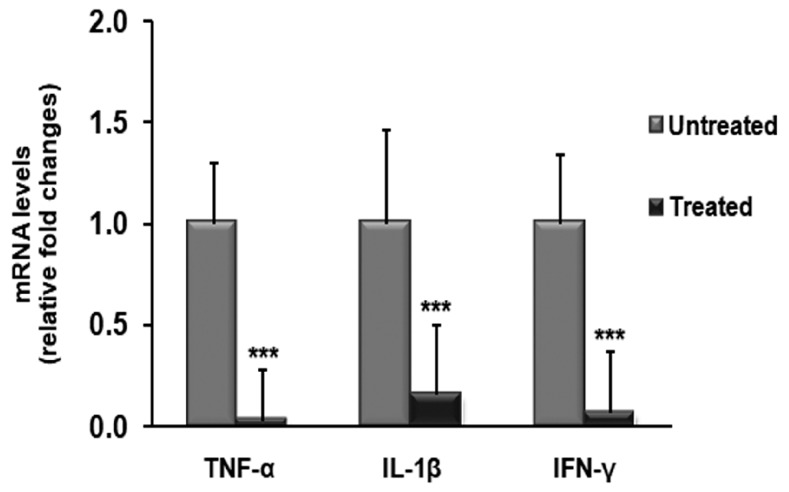
Changes in mRNA levels of cytokines in foreskin tissues from patients with balanitis xerotica obliterans (BXO) untreated or treated with OZOILE^®^. The results are the means of data obtained from 15 untreated patients and 15 treated patients. Error bars represent standard error of the mean (SEM). *** *p* < 0.001 significant differences in comparison with untreated patients.

We found a significant reduction of pro-inflammatory cytokines in foreskin tissues from BXO patients treated with OZOILE^®^ when compared to untreated patients. In particular, mRNA levels of all tested pro-inflammatory cytokines, such as TNF-α, IL-1β and IFN-γ, decreased in OZOILE^®^ treated tissues compared to untreated ones (*p* < 0.001) (Figure 1).

**Figure 2:**
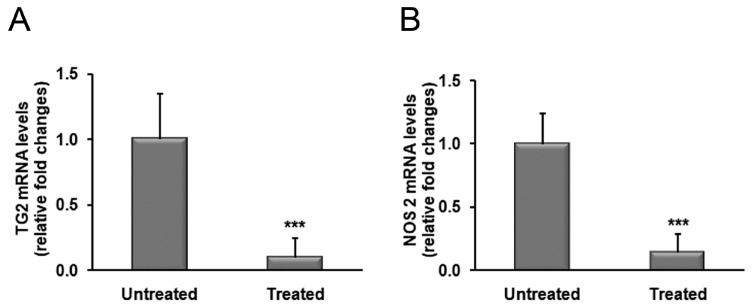
Transcript levels of TG2 (**A**) and NOS2 (**B**) in foreskin tissues from patients with BXO untreated or treated with OZOILE^®^. Results from real-time PCR are expressed as relative fold change compared with foreskin from untreated patients. The data are the means ± standard error of the mean (SEM). *** *p* < 0.001 significant differences in comparison with untreated patients.

The analysis by real-time PCR revealed a significant reduction (~90%) in TG2 transcript levels in patients treated with OZOILE^®^ in comparison to untreated subjects (*p* < 0.001) (Figure 2A). Similarly, the expression of NOS2 was significantly decreased by about 80% in OZOILE^®^ treated tissues compared to untreated foreskins (*p* < 0.001) (Figure 2B).

**Figure 3:**
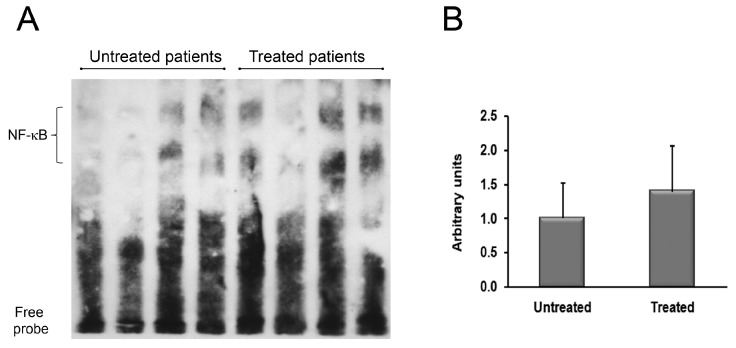
Analysis of NF-κB activation in nuclear extracts from foreskins of BXO patients untreated or treated with OZOILE^®^. DNA binding activity of NF-κB was determined by the electrophoretic mobility shift assay (EMSA). This picture is representative of foreskin tissues from untreated (*n* = 15) and treated (*n* = 15) patients. The densitometric analysis of all samples is also reported. The results are expressed as mean ± SEM.

For a deeper understanding of the molecular mechanisms underlining the anti-inflammatory action of OZOILE^®^ cream, we analyzed the activation status of the transcription factor NF-κB by electrophoretic mobility shift assay (EMSA). As shown in Figure 3, we did not find significant differences in the activation levels of NF-κB between treated and untreated tissues.

**Figure 4:**
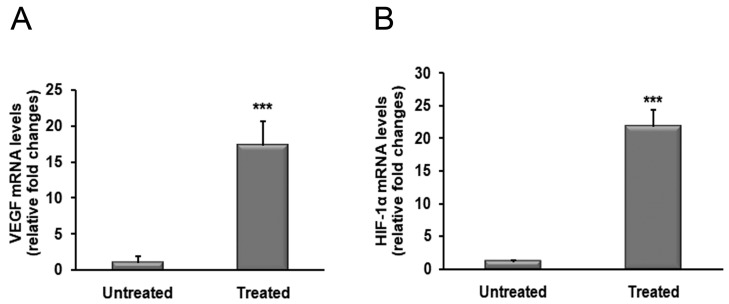
Changes in mRNA levels of VEGF and HIF-1α in foreskin tissues from patients with BXO untreated or treated with OZOILE^®^. The results are the means of data obtained from 15 untreated patients and 15 treated patients. Error bars represent standard error of the mean (SEM). *** *p*< 0.001 significant differences in comparison with untreated patients.

We also analyzed transcript levels of VEGF demonstrating an increase of VEGF in tissues from treated patients in comparison to those from untreated patients. In particular, VEGF mRNA levels were 17-fold higher in BXO patients treated with OZOILE^®^ compared to untreated ones (*p* < 0.001) (Figure 4A).

The possible involvement of HIF-1α in tissue response to OZOILE^®^ was also evaluated, and the results showed an increase by 21 folds in HIF-1α transcript amounts in treated foreskins when compared to untreated tissues (*p* < 0.001) (Figure 4B).

**Figure 5:**
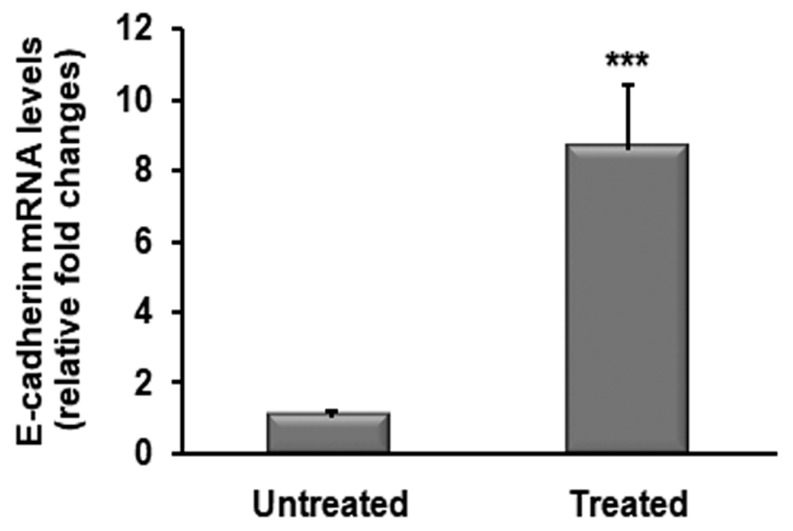
Analysis of expression levels of E-cadherin in foreskins from BXO patients untreated (*n* = 15) or treated (*n* = 15) with OZOILE^®^. Results obtained by real-time PCR are expressed as relative fold change compared with untreated patients. Error bars represent standard error of the mean. *** *p*<0.001 significant differences in comparison with untreated patients.

Furthermore, we analyzed the mRNA levels of E-cadherin and we found that OZOILE^®^ treatment induced an increase in E-cadherin transcript by 8 folds when compared to untreated tissues (*p* < 0.001) (Figure 5).

## 3. Discussion

BXO is a chronic inflammatory dermatosis that occurs on the glans, penis and foreskin and may cause acquired phimosis. It is a chronically progressive process. The cause of BXO disease and the mechanisms of initiation and progression remain largely unknown. Indeed, a frequent association with autoimmune diseases, thyroid disease, alopecia areata, vitiligo, and pernicious anemia has been reported [5,8]. Previous studies demonstrated an association with the risk of gastrointestinal diseases, such as ulcerative colitis, Crohn’s disease, chronic constipation, irritable bowel syndrome [19] and celiac disease [20,21].

Studies in adults have shown an increased incidence of autoantibodies to extracellular matrix protein 1, a glycoprotein which plays an important role in the structural and functional biology of the skin [22]. In addition to autoimmunity, evidence suggesting a genetic link and cases of familial lichen sclerosus [23] as well as an association with different HLA-subtypes [24] have been published. Previous studies, aimed at the characterization of pathogenic processes of BXO, showed an increase in the expression of genes involved in the immune responses of cellular defense [23] and pro-inflammatory processes, and a reduction in proteins essential for tissue remodeling [25] in tissues from patients with BXO compared to healthy tissues.

In our previous study we demonstrated, in male children with BXO, the activation of the inflammatory response characterized by increases in TG2 and IFN-γ expression. Furthermore, alterations of foreskin integrity were evidenced by the reduction of expression of transglutaminase 1 (TG1), transglutaminase 3 (TG3) and E-cadherin [26].

Early diagnosis and treatment lessen the risk of recurrence and meatal/urethral involvement [5]. Meatal stenosis can also cause obstruction of urinary tract [8]. The objectives of BXO treatment are the relieving of symptoms and discomfort and the prevention of meatal or urethral stricture. The recommended treatment is circumcision. Medical treatment can be considered as additional to circumcision and used postoperatively to prevent or reduce complications. In a pediatric age, a study of 36 girl patients affected by LS and treated with high corticosteroid doses showed that 75% of patients had full remission after treatment and only 25% a partial response to treatment, while a study of 40 male children affected by LS showed clinical improvement in 40% [6,27].

On the basis of several study on the topical treatment, a British guideline recommends the initial treatment of boys with BXO with clobetasol proprionate, a highly potent corticosteroid [7]. The use of topical steroids has been proven to arrest or delay the progression of BXO as well as reversing some of the histological changes seen in the disease [28,29]. It has been reported that a treatment with clobetasol propionate (0.05%) for two to three months is successful in over 90% of cases [30]. Ozone therapy represents a versatile bio-oxidative therapy in which oxygen/ozone is administered via gas or dissolved in water or an oil base to obtain therapeutic effects. The treatment is characterized by its excellent curative results, simple application, longer-term effects and non-toxic nature [12,14]. The therapeutic activity of ozonated oil was attributed to its antibacterial, antifungal, antiviral, antiparasitic, antihypoxic, analgesic and immunomodulatory effects on biological systems [12,13,17]. Moreover, the phenomenon of an improved wound healing after the ozone therapy has been related to the rapid changes in cell types and to the release of cytokines modulating the complex healing process [17,31]. Patel and collaborators showed a significant improvement of microbiological and clinical parameters in patients with periodontitis treated with ozonated olive oil in comparison to the control group. Furthermore, in another study on post-operative control of removal of large exophytic gingival lesions, it has been observed an improvement of sign of the inflammation confirmed also by histopathological analysis, as well as a reduction in the surface ulceration [17,18,31].

OZOILE^®^ has been used for treatment of mucosal diseases and healing of the skin. It is a biological inducer capable of modulating the main metabolic pathways by determining cellular and tissue responses restoring damaged functions. Studies have also shown anti-inflammatory and analgesic activities [12,32].

In our observational study, we investigated if the OZOILE^®^ preoperative treatment could bring benefits by reducing the inflammatory process in the diseased foreskin of children with BXO. We examined the mRNA levels of some pro-inflammatory cytokines, IL1-β, TNF-α and IFN-γ in foreskins of children treated with OZOILE^®^ for a week before surgery. Our results demonstrated a powerful anti-inflammatory effect of OZOILE^®^ as showed by the reduction in gene transcript levels of the examined cytokines in treated tissues. Further confirmation has been found upon analysis of TG2 mRNA levels. TG2 is an enzyme with a well-known role in inflammation, and its over-expression has been frequently reported in tissues affected by inflammatory processes [33,34].

In our previous study, an increase in TG2 expression levels in foreskin tissues obtained from BXO untreated patients in comparison to foreskin without BXO has been found. Also, IFN-γ mRNA levels were increased in BXO compared to unaffected foreskin, showing a positive correlation with TG2 transcripts. These results suggested that these proteins may play a role in inducing and maintaining the inflammatory response in foreskins affected by BXO [26]. 

Of note, in the present study we found that OZOILE^®^ treatment was able to reduce the expression levels of TG2, supporting its anti-inflammatory role in BXO affected foreskin.

In regard to the anti-inflammatory activities, we also observed a reduction in the NOS2 mRNA levels after BXO treatment. NOS2, the calcium-independent isoform of the NOS family, produces nitric oxide (NO), an intercellular messenger involved in a wide range of physiological functions, most notably in the control of blood pressure and blood flow [35,36]. However, an increase in NO synthesis is usually associated with the development of pathological conditions [37]. Indeed, NO can rapidly react with other free radicals such as O_2_^−•^ to generate highly reactive oxidant peroxinitrite (ONOO^−^) and other reactive nitrogen species (RNS), which are considered to play a key role in chronic inflammation. The NOS2 expression is generally restricted, but it can be rapidly induced by a variety of stimuli, such as cytokines. Thus, we believe that OZOILE^®^ treatment of foreskin tissues affected by BXO, reducing cytokine production, may lead to the decrease of NOS2 transcription, exerting also beneficial effects against nitrosative stress.

Considering that the expression of both cytokines and TG2 is under the control of NF-κB, a major factor affecting the modulation of the inflammatory response [38], we hypothesized that topical therapy with OZOILE^®^ could exert anti-inflammatory effects by inhibiting NF-κB. 

To sustain this hypothesis, we evaluated the NF-κB activation status in tissue samples obtained from untreated and OZOILE^®^ treated BXO patients. Surprisingly, EMSA analysis did not reveal a substantial change in the activation of this transcription factor between treated and untreated foreskins. Although this result would seem to disagree with the foregoing, it can be explained considering that NF-κB is not the only regulator of cytokines expression [39]. Therefore, other observations could be able to give evidence for involvement of other regulatory elements, including both IKK and cGMP levels.

It has been reported that inflammatory response in patients with BXO is associated with loss of structural integrity of the foreskin [26]. Therefore, we evaluated the effects of OZOILE^®^ treatment in the process of tissue repair of foreskin affected by BXO.

Beneficial effects of ozonated oil in the process of tissue repair have been previously reported [14,40,41,42] and can be associated to its antimicrobial action, but also to its ability to induce the local activation of antioxidant defences and the release of growth factors. Therapeutic doses of ozone produce a moderate oxidative stress which stimulates the expression of numerous antioxidant enzymes by the activation of the nuclear factor-erythroid 2-related factor 2 (Nrf2) [43]. The generation of a moderate pro-oxidant environment leads to the stimulation of molecular mechanisms able to suppress the inflammatory response. Moreover, moderate oxidative stress may induce the expression of the hypoxia-inducible factor-1α (HIF-1α) [44], a transcription factor mainly associated with the adaptive responses of cells to hypoxia [45]. HIF-1α induces the expression of hundreds of genes, among these several growth factors such Vascular endothelial growth factor (VEGF), platelet-derived growth factor (PDGF), fibroblast growth factor (FGF), transforming growth factor-α (TGF-α), and TGF-β [46] all of which are involved in the complex multi-step process of tissue regeneration. VEGF, one of the most potent proangiogenic growth factors in the skin, contributes to blood flow restoration, in this manner providing oxygen, nutrients and other mediators required to support the growth and function of reparative cells in damaged tissues [47].

During tissue repair, cadherin-mediated cell-cell adhesion contributes to the reconstitution of epithelial barrier, and the E-cadherin, a cell-surface glycoprotein belonging to the cadherin family crucial to maintain epithelial integrity [48,49], also participates in tissue re-epithelialization via the modulation of cellular polarity [50], differentiation, growth, and migration [51]. Previously, we reported a loss of E-cadherin in foreskin of subjects affected by BXO in comparison to unaffected tissues. Interestingly, in the present study we demonstrated that OZOILE^®^ treatment is able to up-regulate the expression of E-cadherin and this effect was associated with the overexpression of VEGF and HIF-1α. Based on these observations, we could hypothesize that a moderate oxidative stress elicited by OZOILE^®^ treatment is able to induce the up-regulation of HIF-1α, which in turn may trigger the re-epithelialization process of BXO damaged foreskin by stimulating the initial stages of tissue regeneration such angiogenesis and basement membrane reconstruction.

In our study, we demonstrated that the topical preoperative use of ozonated olive oil with vitamin E acetate cream has a favourable effect on patients undergoing circumcision, inducing a significant reduction of the inflammatory status without adverse reactions. Furthermore, OZOILE^®^ treatment could stimulate a faster recovery and promote the healing process in BXO of children or adolescents undergoing circumcision, suggesting also the possible use of OZOILE^®^ during the postoperative period.

Therefore, our results are encouraging about the treatment of BXO patients with OZOILE^®^, which could be proposed as a good therapeutic alternative to topical corticosteroid, to date the only available therapy for BXO. In addition, given the side effects of corticosteroid, it could be useful a comparison of the effects of OZOILE^®^ cream with other anti-inflammatory drugs, such as corticosteroids.

Nevertheless, in our opinion, a randomized clinical trial on a large number of children affected by BXO might be useful to verify the efficacy of preoperative and postoperative topical treatment with ozonated olive oil with vitamin E acetate.

## 4. Materials and Methods

### 4.1. Patient Recruitment

Thirty children undergoing circumcision and with histological diagnosis of BXO were included in this observational study. The mean age at diagnosis was 9.9 ± 3.3 years, ranging from 5 to 15 years. All the patients showed no alteration in the blood count or c reactive protein at the time of surgery. Informed consent and authorization to use sensitive data were obtained from the parents of all subjects at admission. The study was approved by the ethical committee of our hospital and all procedures performed in this study were in accordance with the ethical standards of the institutional and/or national research committee and with the 1964 Helsinki declaration and its later amendments or comparable ethical standards.

The histological diagnosis of BXO was defined by pathologists as an epithelial-stromal lesion characterized by squamous atrophy or hyperplasia, band like infiltration, hyalinization of the papillar dermis, hyperkeratosis, pigment incontinence and/or dermal edema.

Before circumcision, 15 patients with BXO were treated by pediatrician with OZOILE^®^ cream once a day for 7 days, parallelly fifteen age-matched patients affected by BXO, but without any treatment, were recruited as control group. Clinically diseased foreskin samples were obtained during circumcision procedure from patients with and without treatment. The samples were divided into two parts: one portion submitted for histological analysis and the other for the analysis of transcript levels of pro-inflammatory cytokines, transglutaminase 2 (TG2), inducible nitric oxide synthase (NOS2), hypoxia inducible factor 1α (HIF-1α), vascular endothelial growth factor (VEGF) and E-cadherin by quantitative real-time PCR, as well as for the evaluation of NF-κB activation status by electrophoretic mobility shift assay.

### 4.2. Gene Expression Analysis

After sampling, foreskin tissues were immersed in 500 µL of RNA stabilization reagent (RNAlater; Life Technologies, Milan, Italy), and stored at −80 °C until RNA was isolated. Total RNA was isolated using TRIzol reagent (Life Technologies), and two micrograms of total RNA were reverse-transcribed with high-capacity cDNA Archive kit (Life Technologies). Then, mRNA levels of TNF-α, IL-1β, IFN-γ, TG2, NOS2, HIF-1α, VEGF and E-cadherin were analyzed by Sybr Green real-time PCR.

Quantitative PCR reactions were set up in duplicate in a 96-well plate and were carried out in 10 µL reaction volume containing 1×Sybr Green Master Mix (Life Technologies), 0.1 µM specific primers and 25 ng RNA converted into cDNA. Real-time PCR was performed in a 7900HT fast real-time PCR system (Applied Biosystems, Foster City, CA, USA) with the following profile: one cycle at 95 °C for 10 min, followed by 40 cycles at 95 °C for 15 s and 60 °C for 1 min. A standard dissociation stage was added to assess primer specificity.

Data were analyzed using the 2^−^^ΔΔ^^Ct^ relative quantification method, and values are presented as fold change relative to untreated group. β-actin was used as endogenous control.

The primer sequences are reported in Table 1.

### 4.3. EMSA (Electrophoretic Mobility Shift Assay)

The presence of NF-κB DNA binding activity in nuclear extracts was evaluated by EMSA as described by Caccamo et al. [52]. To prepare nuclear extracts, after treatment cells were homogenized in ice-cold hypotonic lysis buffer (10 mM HEPES, pH 7.9, 0.1 mM EGTA, 0.1 mM EDTA, 0.1 mM dithiothreitol, 50 mM sodium fluoride, 1 mM sodium orthovanadate, 30 mM β-glycerophoshate, 20 mM*p*-nitrophenyl phosphate, 0.5 mM phenylmethylsulfonyl fluoride, 1% NP-40 and 10 µg/mL each of aprotinin, leupeptin, and pepstatin). Samples were incubated for 20 min on ice and then centrifuged at 2000 rpm for 2 min at 4 °C to sediment membranes. The supernatants were centrifuged at 10,000 rpm for 1 min at 4 °C to sediment nuclei, which were resuspended in ice-cold hypertonic nuclear extraction buffer (20 mM HEPES, pH 7.9, 420 mM NaCl, 1 mM dithiothreitol, 1 mM EDTA, 50 mM sodium fluoride, 1 mM sodium orthovanadate, 30 mM b-glycerophosphate, 0.5 mM phenylmethylsulfonyl fluoride, 20 mM*p*-nitrophenylphosphate, and 10 µg/mL each of aprotinin, leupeptin and pepstatin), incubated on ice for 30 min with intermittent vortexing, and centrifuged at 10,000 rpm for 10 min at 4 °C. The supernatant was collected as nuclear extract, and protein concentration was determined by Bradford assay.

Nuclear proteins (2 μg) were incubated with the double-stranded biotin-labeled NF-κB DNA probe, and then the protein/DNA complexes were separated on a non-denaturing 6% polyacrylamide gel. After transferring onto a nylon membrane, detection was performed using streptavidin-HRP and a chemiluminescent substrate. The membrane was developed by exposure to film and the bands were scanned and quantified by densitometric analysis with ImageJ 1.47, an open source software freely downloadable from the US National Institute of Health website (http://imagej.nih.gov/ij/).

### 4.4. Statistical Analysis

All values are expressed as mean ± standard error of the mean (SEM). Statistical analysis of gene and protein expression data was carried out using Student’s *t*-test for comparisons between two groups, with *p*-values < 0.05 considered significant.

## Figures and Tables

**Table 1:** qRT-PCR primer sequences.

Gene	Forward Primer (5′→3′)	Reverse Primer (5′→3′)
*ACT-β*	TGGTTACAGGAAGTCCCTTGCC	ATGCTATCACCTCCCCTGTGTG
*IL1-β*	GCTTATTACAGTGGCAATGA	TAGTGGTGGTCGGAGATT
*TNF-α*	GTGAGGAGGACGAACATC	GAGCCAGAAGAGGTTGAG
*IFN-γ*	GCAGCCAACCTAAGCAAGAT	TCACCTGACACATTCAAGTTCTG
*TG2*	CCTTACGGAGTCCAACCTCA	CCGTCTTCTGCTCCTCAGTC
*NOS2*	TGACCTCCTAACAAGTAGCA	CAGCAGCAAGTTCCATCT
*HIF-1α*	CGTTCCTTCGATCAGTTGTC	TCAGTGGTGGCAGTGGTAGT
*VEGF*	AGGAGGAGGGCAGAATCATCA	CTCGATTGGATGGCAGTAGCT
*E-cadherin*	TGAGTGTCCCCCGGTATCTTC	CAGTATCAGCCGCTTTCAGATTTT
